# Efficient Production of High-Quality Few-Layer Graphene Using a Simple Hydrodynamic-Assisted Exfoliation Method

**DOI:** 10.1186/s11671-018-2830-9

**Published:** 2018-12-27

**Authors:** Zhiliang Zhang, Hailun Jin, Chenyu Wu, Jianbing Ji

**Affiliations:** 1 College of Chemical Engineering, Zhejiang University of Technology, Hangzhou, 310014 China; 2 Zhejiang Province Key Laboratory of Biofuel, Biodiesel Laboratory of China Petroleum and Chemical Industry Federation, Zhejiang University of Technology, Hangzhou, 310014 China

**Keywords:** High-quality few-layer graphene, Liquid phase exfoliation, Needle valve, Hydrodynamic-assisted

## Abstract

Graphene, a two-dimensional nanomaterial, has shown tremendous promising applications in a broad range of fields. Mass production of defect-free graphene is a prerequisite for its applications. In this work, by using a needle valve, we propose a simple hydrodynamic-assisted exfoliation method to produce high-quality few-layer graphene flakes. The prepared graphene flakes, with an average layer of 5 (~ 71% less than five layers) and a Raman D/G intensity ratio as low as 0.1, are free of defects and oxidation. The average thickness and length of the few-layer graphene flakes are 2.3 nm (~ 90% < 4 nm) and 1.9 μm (~ 50% in the range of 1–7 μm), respectively. In a lab-scale trial, the concentration of graphene can reach 0.40 g/ml under mild operating conditions (working pressure 20 MPa, 16 cycles), and the corresponding production rate is 0.40 g/h. The hydrodynamic-assisted exfoliation by needle valve potentially offers a simple and efficient method for large-scale production of high-quality graphene.

## Introduction

Graphene, a single layer of graphite, has been attracting growing attention since its discovery in 2004 [1]. Owning to its impressive physical and chemical properties [2], graphene has shown tremendous promising applications in a broad range of fields, such as electronics [3], photonics [4], catalysis [5, 6], energy conversion/storage [7–9], and polymer nanocomposites [10, 11]. To fulfill these exciting potential applications, particular attention has been drawn to the production of high-quality graphene on large-scale.

To date, many methods such as micromechanical cleavage [12], chemical vapor deposition [13, 14], solvothermal synthesis [15], chemical exfoliation [16, 17], and liquid phase exfoliation [18, 19] have been proposed to produce graphene. Among these, liquid phase exfoliation, namely exfoliation of graphite for preparation of graphene in a liquid media, is considered to be one of the most promising and simplest approaches to achieve mass production of graphene at low cost [19]. Liquid phase exfoliation is usually implemented by ultrasonication. However, the ultrasonic exfoliation is highly dependent on the geometry of ultrasonic vessel size and shape, which makes this method possess low yield, time consuming, and particularly, impossibility of scale-up [20, 21]. In addition, recent studies indicated that the graphene produced by ultrasonic exfoliation has many more structure defects than expected [22].

Recently, as an alternative pathway, fluid dynamics-assisted liquid phase exfoliation, has been proposed to produce graphene with low defect content on large-scale [21, 23–33]. By using a jet cavitation device, Liang et al. [29] prepared a series of graphene dispersions with the maximum concentration of 0.12 mg/ml, whereas the processing time was long up to 8 h. Liu et al. [26] produced a graphene dispersion with the concentration of 0.27 mg/ml using a specially designed high shear mixer. Nacken et al. [31] showed the production of graphene dispersion with the concentration of 0.223 mg/ml by a high pressure homogenizer. Yi et al. [27] demonstrated the feasibility of exfoliation by a kitchen blender, and graphene with the concentration of 0.22 mg/ml was produced. Using the kitchen blender, a higher concentration of 1 mg/ml was achieved by Varrla et al. [30]. Previous studies have shown that the fluid dynamics-assisted exfoliation has a good prospect for scalable production of graphene. However, because intensive operating conditions and long processing time are usually required for this technique, the obtained graphene are characterized by high Raman D/G intensity ratios (*I*_D_/*I*_G_, a measure of defect content) and low aspect ratios. For example, Liang et al. [29] reported an *I*_D_/*I*_G_ value of 0.38 for jet cavitation exfoliated graphene, while the length was unknown. The graphene produced by kitchen blender (*I*_D_/*I*_G_ = 0.3–0.7, length = 0.63 μm) and high-pressure homogenizer (*I*_D_/*I*_G_ = 0.52–0.78, length = 0.02–0.58 μm) were also featured with high *I*_D_/*I*_G_ values and low aspect ratios [30, 31]. Different fluid dynamics-assisted exfoliation methods give *I*_D_/*I*_G_ and length in the range of 0.14–0.78 and 0.02–1.41 μm [26–33], respectively. Therefore, a more efficient method in terms of both higher graphene concentration and high aspect ratio is of great significance.

In this work, a simple method based on hydrodynamic mechanism was proposed for scalable production of high-quality few-layer graphene flakes. A simple needle valve was used as exfoliation device. The exfoliation process was exemplified using 80 wt% *N*-methyl pyrrolidone aqueous solution as solvent [34, 35]. Quality of the products were characterized by scanning electron microscopy (SEM), transmission electron microscopy (TEM), atomic force microscopy (AFM), Raman spectroscopy, and X-ray photoelectron spectroscopy (XPS). The effects of operating parameters on graphene concentrations were also investigated.

## Materials and Methods

### Materials

*N*-methyl pyrrolidone (NMP) (purity 99.5%) and graphite powder (≤ 325 mesh, purity 99.9%) were purchased from Aladdin Industrial Corporation in Shanghai (China). Deionized water was purified by a laboratory water purification system (SZ-97A, Shanghai, China).

### Exfoliation of Graphite into Few-Layer Graphene Flakes

**Fig. 1:**
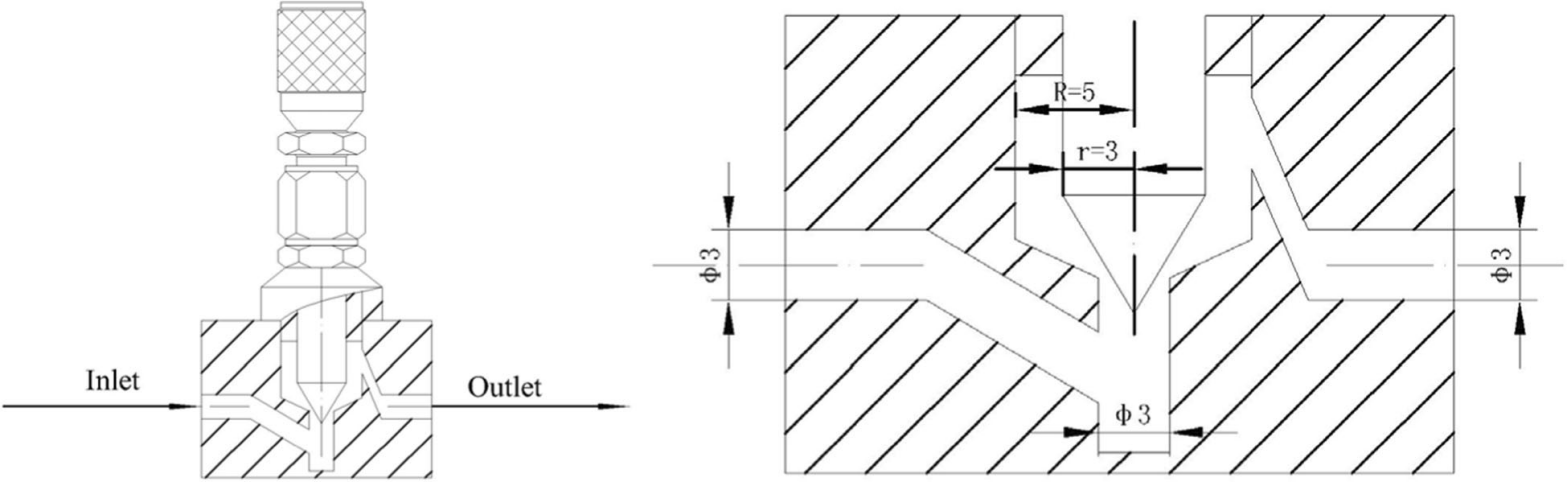
A schematic view of the needle valve

**Fig. 2:**
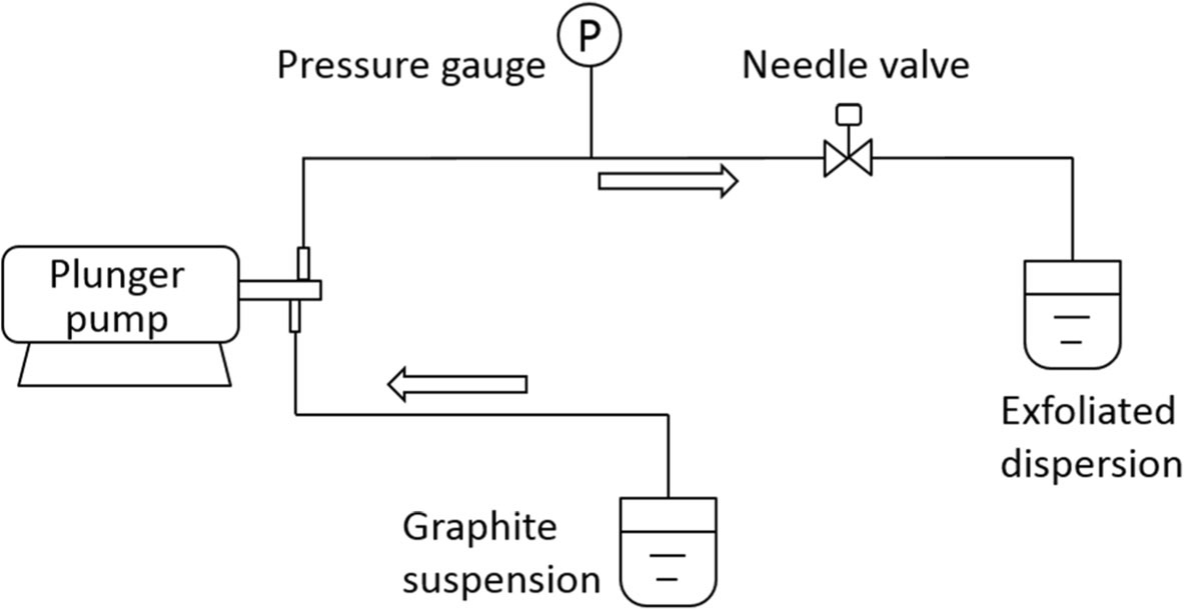
Schematic diagram of the hydrodynamic-assisted exfoliation process

A schematic view of the needle valve used for exfoliation is shown in Fig. 1. When a liquid passes through the narrow gap in the valve, cavitation and velocity gradient can be generated due to abrupt velocity and geometrical change, which may induce normal force and shear force for exfoliation. By simply adjusting the width of the valve gap, the working conditions can be varied and controlled. Figure 2 shows a flow diagram of the hydrodynamic-assisted exfoliation process by needle valve. In a typical experiment, graphite powder was dispersed in 80 wt% NMP aqueous solution to obtain graphite suspension with the concentration of 10 mg/ml. Then, the suspension was pumped by a plunger pump (model 2-JW, Zhijiang Petrochemical, China) through the needle valve. By adjusting the opening of the valve, the working pressure was controlled at 20 MPa. After 16 cycles, the dispersion was collected and subsequently centrifuged at 500 rpm for 60 min (SC-3610, USTC Zonkia, China) to remove unexfoliated graphite. Subsequently, the supernatant dispersion was decanted and retained for further use.

### Characterization

The morphology and size of graphene were analyzed by SEM (VEGA3, TESCAN). Samples for SEM were coated with gold in an argon atmosphere. TEM was performed by a Tecnai G2 F30 S-Twin and operated at 300 kV. The samples were prepared by dropping the graphene dispersion onto holey carbon grids. AFM images were captured in tapping mode using a Bruker Dimension Icon. A newly cleaved mica was used as substrate for AFM analysis. Raman spectroscopy was conducted with a Lab RAM HR800 (λ = 532 nm) at room temperature. XPS was employed to detect the oxidation defect of graphene using an ESCALAB 250Xi analyzer. UV-Vis absorption was performed to measure graphene concentration by a Lambda 35 spectrophotometer (PerkinElmer) at a wavelength of 660 nm.

## Results and Discussion

### Quality of Graphene Flakes

**Fig. 3:**
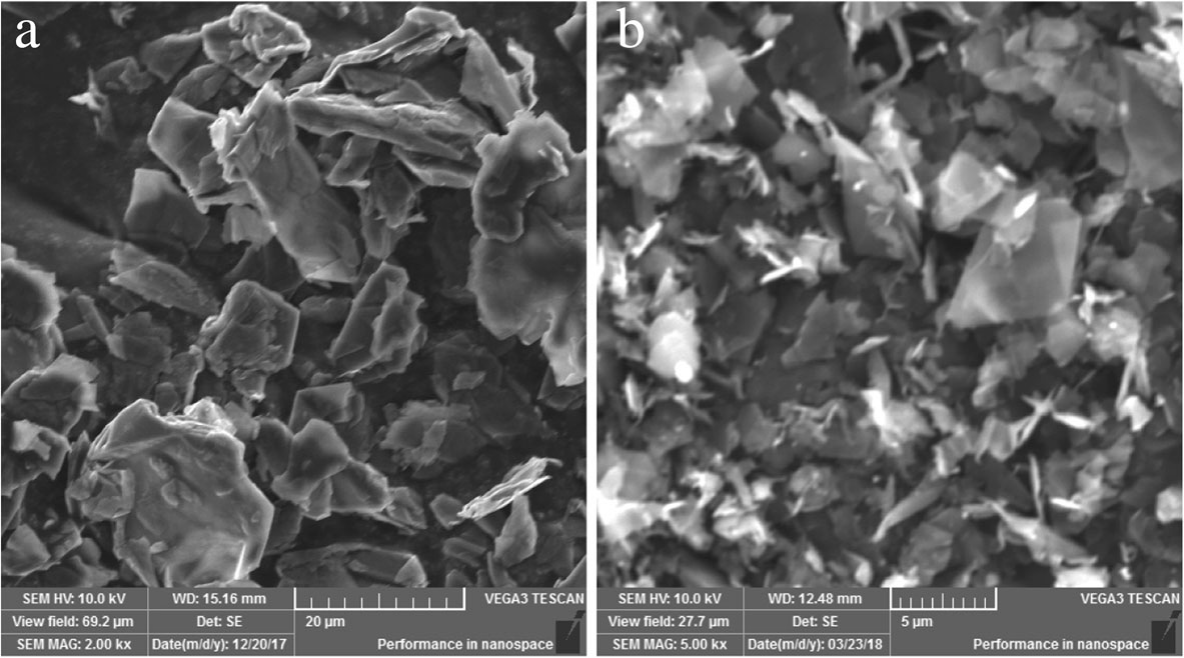
SEM images of **a** the bulk graphite and **b** the prepared graphene powder

Figure 3 displays typical SEM images of the bulk graphite and the prepared graphene powder. The bulk graphite was flake-like powder with a lateral size and a thickness of approximately 5–20 μm and 10 μm, respectively. In comparison, the prepared graphene powder contains considerably thinner flakes with a lateral size that decreased to approximately 1–7 μm, while the thickness was far below 1 μm. Clearly, the bulk graphite was exfoliated into small flakes. Note that several flakes with folded edges were appeared, which were believed as mono-layer or few-layer graphene flakes [26].

**Fig. 4:**
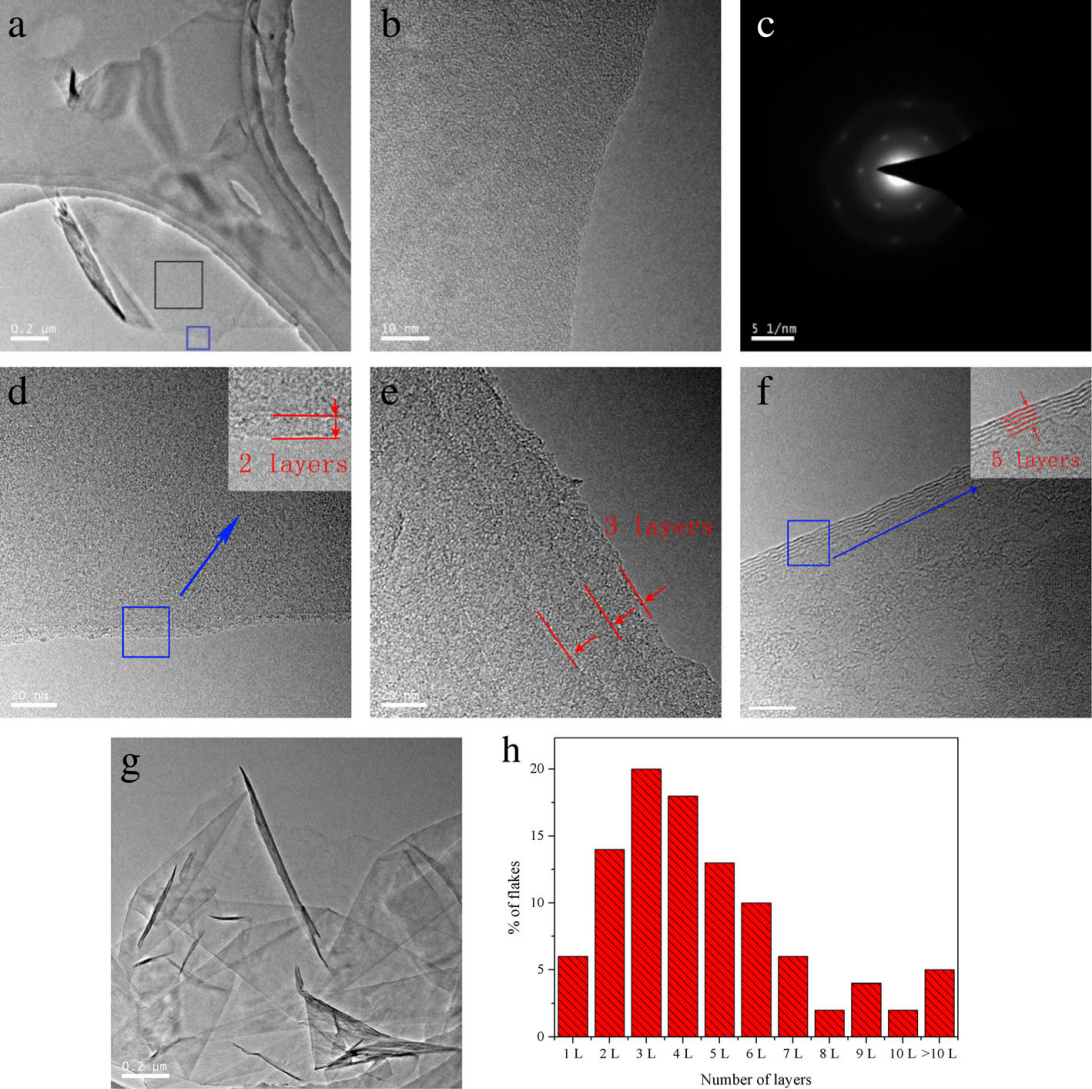
Typical TEM images and electron diffraction of the prepared graphene flakes. **a** Mono-layer graphene flake with folded edge, **b** magnified image of the blue box in image (**a**), **c** electron diffraction of the selected black box in image (**a**), **d** a bilayer graphene flake, **e** a trilayer graphene flake, **f** a five-layer graphene flake, **g** several individual graphene flakes, **h** distribution of number of layers (obtained from TEM analysis of at least 100 graphene flakes)

To identify the formation of mono-layer or few-layer graphene, TEM analysis was performed to visually observe the number of graphene layers. Figure 4 presents typical TEM images of the prepared graphene flakes. Figure 4a shows a mono-layer graphene flake with folded edge. A high-resolution TEM image of the blue box in Fig. 4a is displayed in Fig. 4b. Smooth edge that dominated by one dark line was observed clearly, indicating the formation of mono-layer graphene [23]. A more definitive identification of mono-layer graphene was further confirmed by selected-area electron diffraction patterns (selected from the black box in Fig. 4a). As shown in Fig. 4c, a typical diffraction of mono-layer graphene was presented, that is, the inner spots {1100} were more intense than the outer spots {2110} [18, 36, 37]. The hexagonal diffraction pattern indicates a good crystallinity of the prepared graphene [18]. Figure 4d–f are typical TEM images of bilayer, trilayer, and five-layer graphene flakes. Figure 4g is an image of several individual graphene flakes stack together due to the tendency of agglomeration. A statistical analysis of the layer distribution was obtained from TEM analysis of at least 100 graphene flakes. As shown in Fig. 4h, ~ 71% of the flakes were less than five layers, and the average layer was 5, indicating a high quality of graphene. Note, due to the fact that graphene flakes with very small size would be lost through the holey TEM grids, the statistical results of layer were probably higher than the corresponding actual value.

**Fig. 5:**
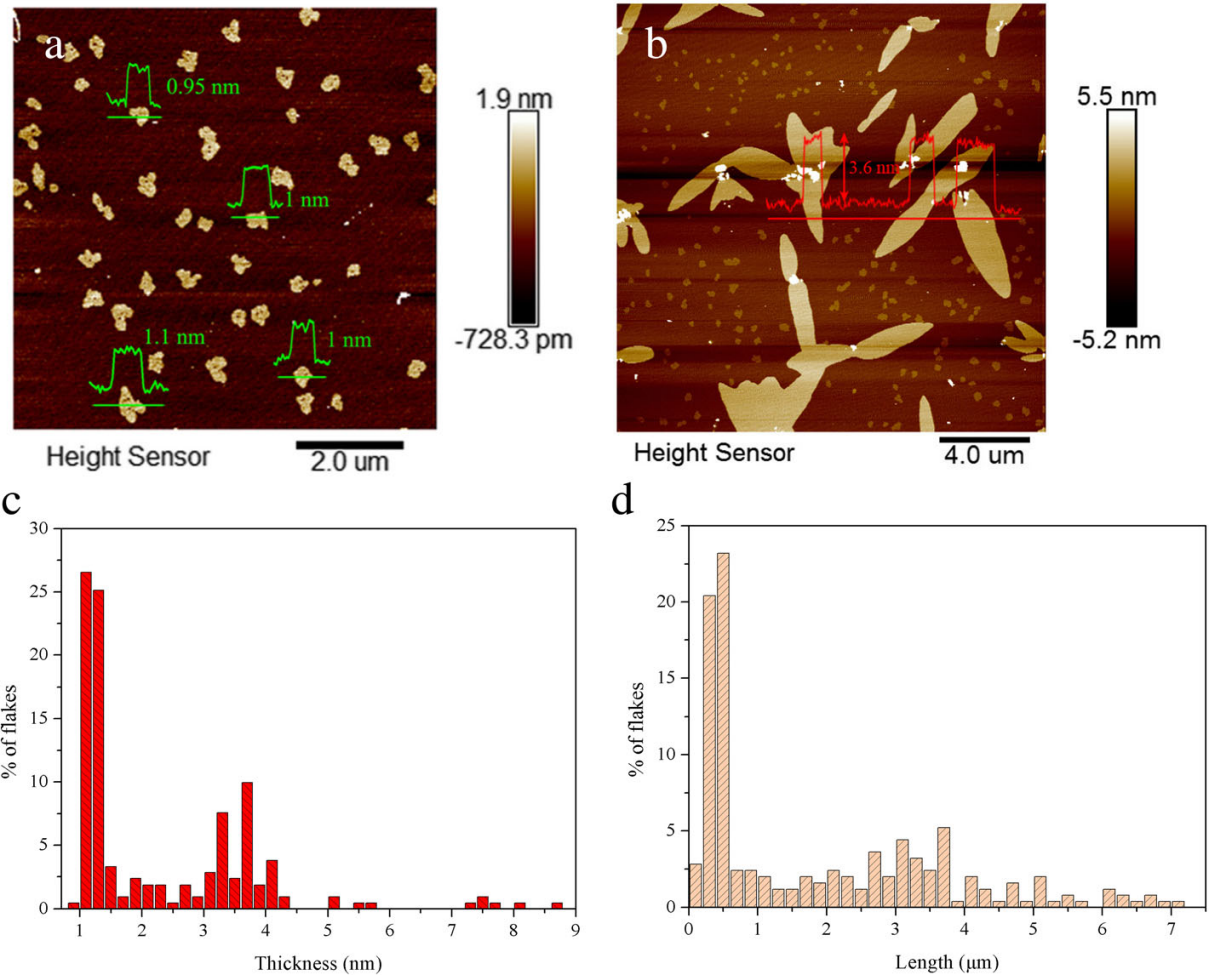
Representative AFM images of **a** mono-layer graphene flakes and the corresponding height profiles, **b** few-layer graphene flakes and the corresponding height profiles, **c** thickness distribution of flakes, and **d** length distribution of flakes (**c** and **d** were obtained from AFM analysis of at least 200 graphene flakes)

To further identify the thickness and length of graphene, AFM analysis was performed by using mica wafer as the substrate. Shown in Fig. 5a is a typical AFM image of mono-layer graphene flakes. The cross-sectional analysis indicated that the topographic height of the flakes is approximately 1 nm, which could be considered as mono-layer flakes according to the fact that the mono-layer graphene is usually measured to be 0.4–1 nm by AFM due to the analysis equipment and substrates and residual water [38]. Few-layer graphene flakes could be observed from Fig. 5b. The thickness of these flakes was ~ 3.6 nm, while the length was as high as 3–5 μm. Further statistical analyses of the thickness/length distributions were obtained from AFM analysis of at least 200 graphene flakes. As shown in Fig. 5c, d, ~ 90% of the graphene flakes were less than 4 nm. Only a minority of ~ 5% flakes with the thickness of more than 5 nm were observed. Further, ~ 50% of the flakes had the length in the range of 1–7 μm. The average thickness and length of the flakes were 2.3 nm and 1.9 μm, respectively, confirming the high quality of graphene.

**Fig. 6:**
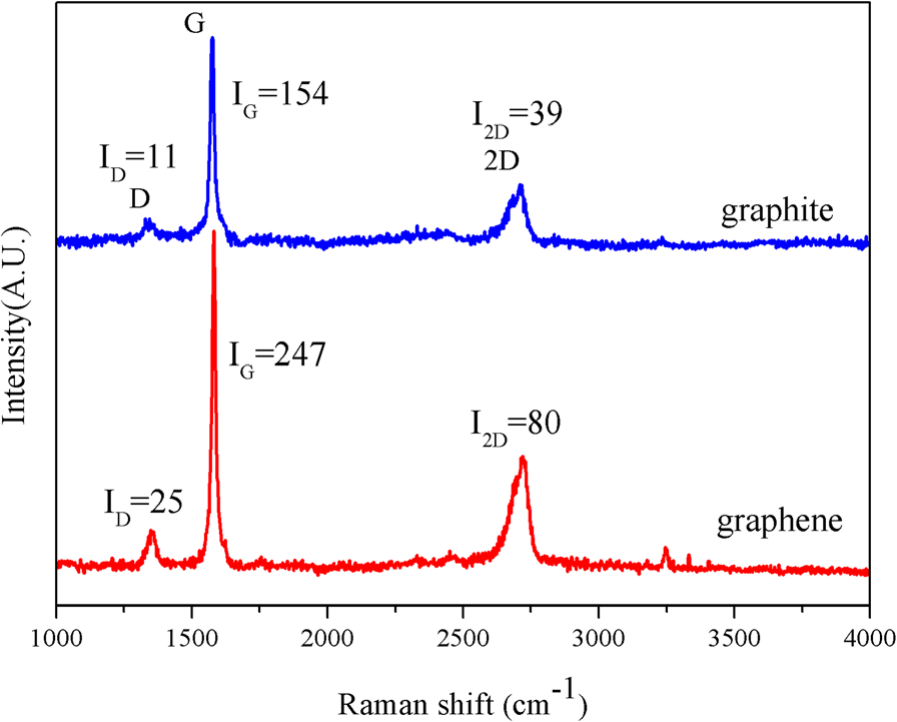
Raman spectroscopy of the bulk graphite and graphene

Raman spectroscopy was performed to detect the defect content of graphene. Figure 6 shows typical Raman spectra of the prepared graphene along with the bulk graphite as a reference sample. Three characteristic peak, i.e., D band (~ 1350 cm^−1^), G band (~ 1580 cm^−1^), and 2D band (~ 2700 cm^−1^) were observed for these two graphitic materials. For graphene, the 2D band was in the shape of symmetric peak. The full width at half maximum (FWHM) of G band was 13 cm^−1^, well matching with the previous reports for thin graphene flakes (12–14 cm^−1^) [39]. Moreover, the intensity ratio of D/G (*I*_D_/*I*_G_) for the prepared graphene was 0.10, lower than that of ultrasonication exfoliated graphene (0.29) [32], shear force exfoliated graphene (0.17–0.37) [24, 26], and other fluid dynamics exfoliated graphene (0.21–0.78) [31, 32], further verifying the high quality of graphene.

**Fig. 7:**
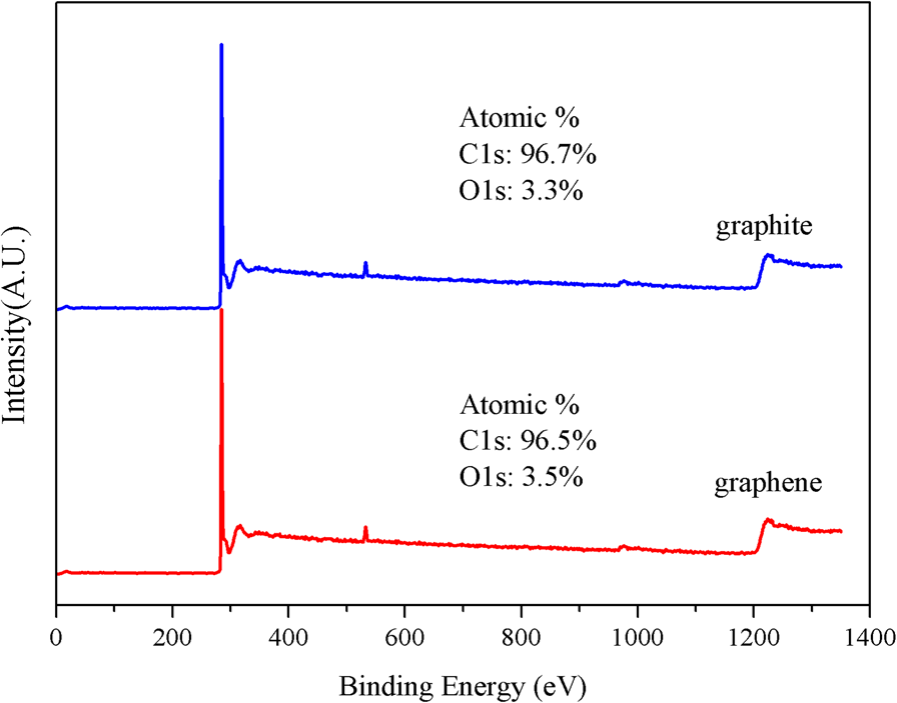
XPS spectra of the bulk graphite and graphene

The oxides content of the prepared graphene flakes was investigated by XPS. As shown in Fig. 7, the XPS spectra of the prepared graphene displayed the same bonds and similar composition with that of the bulk graphite, indicating the absence of chemical modification or oxidization during the exfoliation process. The above results demonstrated that hydrodynamic-assisted exfoliation by needle valve is an efficient method to produce unoxidized few-layer graphene with high quality.

### Effects of Operating Parameters on the Concentration of Graphene

To maximize the productivity of few-layer graphene, the effects of operating parameters, that is, working pressure (*P*), number of cycles (*N*), and initial concentration of bulk graphite (*C*_i_), on the concentration of few-layer graphene dispersion were investigated.

**Fig. 8:**
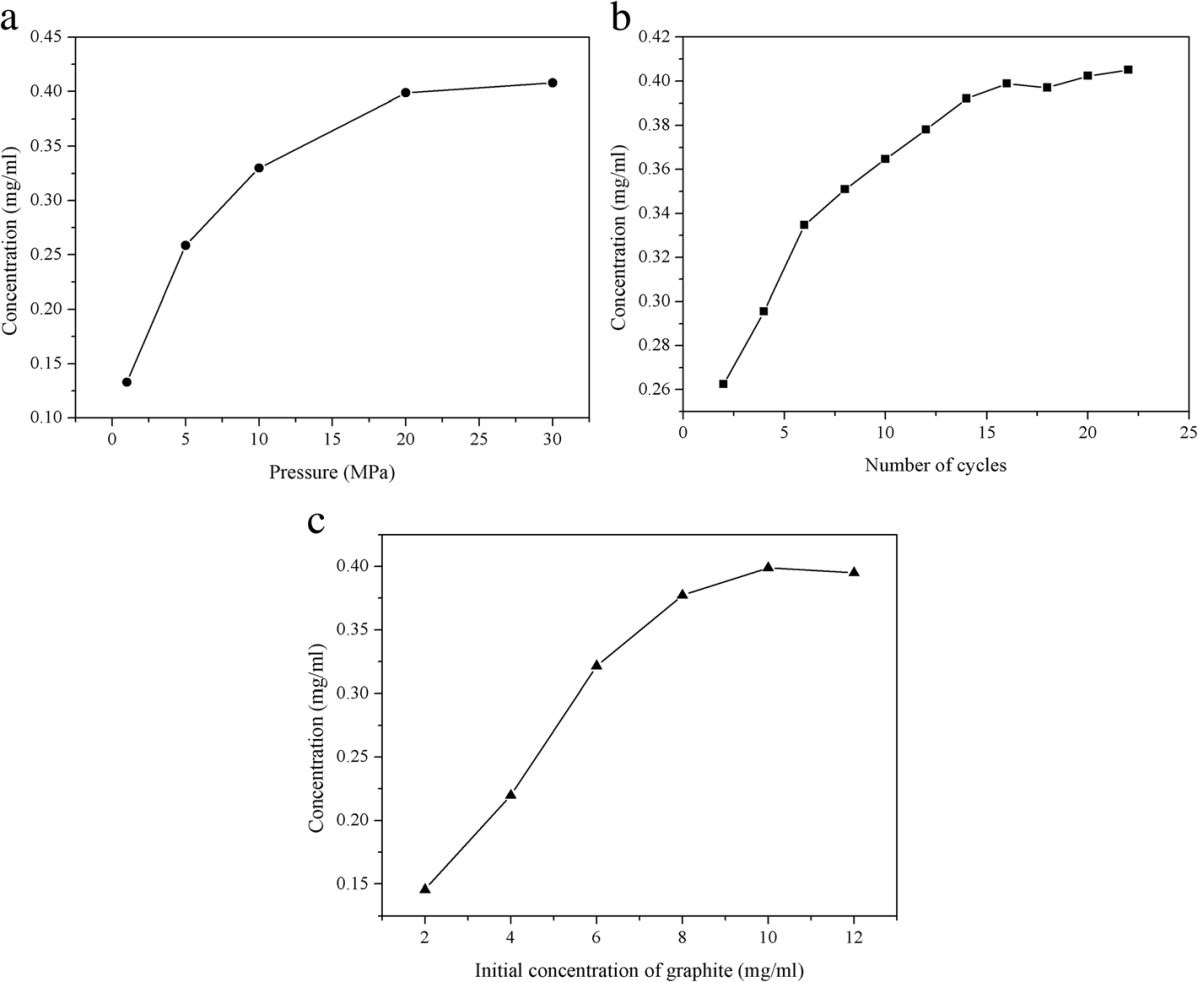
Effects of operating conditions on the concentration of few-layer graphene. **a** Working pressure, **b** number of cycles, and **c** initial concentration of graphite

As shown in Fig. 8a (*N* = 16, *C*_i_ = 10 mg/ml), the concentration obviously increased with increasing working pressure from 1 to 20 MPa, and a concentration as high as 0.40 mg/ml was reached at 20 MPa. However, further increasing the working pressure to 30 MPa, no significant increase in the concentration was observed. Such results may be explained by the following reasons. In hydrodynamic-assisted exfoliation process, increasing in the working pressure results in an increase in the collapse intensity of cavity, due to which there is an increase in the magnitude of the stress derived from cavitation and turbulent, thereby favoring the delamination of graphite. The concentration did not change appreciably above 20 MPa, probably due to the agglomeration and re-stacking of graphene flakes caused by the rise of temperature under higher working pressure [31].

Figure 8b shows the dependence of number of cycles on graphene concentration (*P* = 20 MPa, *C*_i_ = 10 mg/ml). As expected, the concentration was increased with increasing number of cycles. With 16 cycles, the concentration reached a maximum value of 0.4 mg/ml. However, further increasing the number of cycles, the concentration was basically unchanged. In the exploitation process by needle valve, passing the suspension through the valve more than once could result in the fragmentation of graphite, which was favorable for exploitation due to the fact that smaller graphite pieces are easier to delaminate into graphene than larger ones [25]. However, as the graphite flakes become smaller, the size of graphite was comparable with that of cavitation bubbles [39]. Therefore, the formation of new graphene flakes becomes difficult.

Initial concentration of bulk graphite also had a significant influence on graphene concentration [40]. As shown in Fig. 8c (*P* = 20 MPa, *N* = 16), the concentration increased from 0.146 to 0.40 mg/ml with increasing the initial concentration of graphite from 2 to 10 mg/ml. In liquid phase exfoliation process, particle-particle collisions are beneficial to the delamination of graphite. A higher initial concentration of graphite leads to the reinforcement of particle collision, thereby contributes to the self-exfoliation of graphene [26]. When the initial concentration of graphite further increased to 12 mg/ml, the graphene concentration decreased slightly. Similar results were observed by Liang et al. [29] and Arao et al. [32], indicated that over-concentrated graphite dispersion would hinder instead of promote graphene concentration.

### Comparison of Few-Layer Graphene Produced by Different Fluid Dynamics Methods

Table 1 presents a summary of the concentration, length, and *I*_D_/*I*_G_ of the few-layer graphene produced by different fluid dynamics methods. The graphene produced by needle valve had a concentration as high as 0.40 mg/ml, which was higher than most of the reported values. In a lab-scale trial, the production rate was calculated to be 0.40 g/h. The concentrations reported by Varrla et al. [30] and Arao et al. [32] could reach up to 1 mg/ml and 7 mg/ml, respectively. However, the length of their products was smaller (0.63 μm, 1.41 μm). In contrast, the graphene obtained in this study had an average length of 1.9 μm (larger than the reported sizes) and Raman *I*_D_/*I*_G_ as low as 0.1 (lower than the reported ratios). Therefore, it can be concluded that the hydrodynamic-assisted exfoliation by needle valve was an efficient approach to produce few-layer graphene flakes with high quality.

**Table 1:** A summary of the concentration, length, and *I*_D_/*I*_G_ of the few-layer graphene produced by different fluid dynamics methods

Methods	Graphene concentration	Length	Raman *I*_D_/*I*_G_	Operating conditions	Reference
Needle valve	0.4 mg/ml	1.9 μm	0.1	Pressure: 20 MPa	This work
Jet cavitation	0.12 mg/ml	–	0.277	Pressure: 30 MPa	Liang et al. [29]
High shear mixer	0.27 mg/ml	0.35–0.9 μm	0.14–0.18	Rotor speed: 9500 rpm	Liu et al. [26]
High shear mixer	0.07 mg/ml	0.3–0.8 μm	0.17–0.37	Rotor speed: 4500 rpm	Paton et al. [28]
Shear mixer	0.0576 mg/ml	0.35 μm	0.25–0.63	Rotor speed: 1000 rpm	Xu et al. [33]
High pressure homogenizer	0.223 mg/ ml	0.02–0.58 μm	0.52–0.78	Pressure: 53 MPa	Nacken et al. [31]
High pressure homogenizer	7 mg/ ml	1.41 μm	0.21	Pressure: 50 MPa	Arao et al. [32]
High pressure homogenizer	–	0.31 μm	0.24	Pressure: 35–43 MPa	Arao et al. [35]
Kitchen blender	0.22 mg/ ml	–	< 0.12	Rotor speed: 5000 rpm	Yi et al. [27]
Kitchen blender	1 mg/ml	0.63 μm	0.3–0.7	Rotor speed: 18,000 rpm	Varrla et al. [30]

### Possible Exfoliation Mechanisms

We suggest the superiority of the hydrodynamic-assisted exfoliation be ascribed to the exfoliation mechanism. Considering the structure of the needle valve, the flowing fluid dynamics effects are responsible for the delamination of graphite: First, when the suspension containing graphite passes through the narrow gap of the valve, the total pressure of the liquid falls sharply below its vapor pressure. As a result, a turbulent jet that causes huge hydrodynamic stress is formed at the outlet of the valve and generating a large volume of cavitation bubbles. The bubbles then grow from micro gas nucleuses and subsequently collapse intensely. With the collapse of bubbles, intensive microjets and shock waves that surround the graphite are generated, thus resulting in the delamination [41]. Second, the velocity gradient and collision also contribute to the exfoliation. When liquid jets out from the narrow gap of the valve, viscous shear force, which is preferable for the delamination, can be induced by velocity gradient due to abrupt velocity and geometrical change [32]. In addition, the self-exfoliation of graphene caused by the collision of graphite particles is also favorable for the exfoliation [26].

## Conclusions

In summary, we have demonstrated the production of high-quality few-layer graphene using a simple hydrodynamic-assisted exfoliation method. The results indicated that ~ 71% of the prepared graphene flakes were less than five layers, while the average thickness and length of the flakes were 2.3 nm (~ 90% < 4 nm) and 1.9 μm (~ 50% in the range of 1–7 μm), respectively. The flakes with *I*_D_/*I*_G_ ratio as low as 0.1 were free of defects and oxidation. In a lab-scale trial, the concentration of few-layer graphene could reach 0.40 mg/ml under mild operating conditions (working pressure 20 MPa, 16 cycles), and the corresponding production rate was 0.40 g/h. Hydrodynamic-assisted exfoliation by needle valve was potentially an efficient method for scalable production of high-quality graphene.

## Abbreviations

AFM: Atomic force microscopy
*C*_i_: Initial concentration of bulk graphite
FWHM: Full width at half maximum
*I*_D_/*I*_G_: Raman D/G intensity ratio
*N*: Number of cycles
NMP: *N*-methyl pyrrolidone
*P*: Working pressure
SEM: Scanning electron microscopy
TEM: Transmission electron microscopy
XPS: X-ray photoelectron spectroscopy
